# From frailty to decision: impact of comprehensive geriatric assessment on surgical planning and outcomes in older adults with gastrointestinal malignancies

**DOI:** 10.3389/fonc.2026.1788002

**Published:** 2026-04-13

**Authors:** Ran Orgad, Mohammad Igbariya, Nadav Y. Schacham, Saeed M. Naser, Niv Hagai, Adva Kuper, Laura N. Frain, Hanoch Kashtan, Lisa Cooper

**Affiliations:** 1 Department of Surgery, Assuta Ashdod Hospital, Ashdod, Israel; 2 Department of Geriatric Medicine, Rabin Medical Center, Petah Tikva, Israel; 3 Brigham and Women’s Hospital Division of Aging, Boston, MA, United States

**Keywords:** comprehensive geriatric assessment, frailty, geriatric surgery, prehabilitation, shared decision-making, surgical oncology, treatment planning

## Abstract

**Background:**

Frailty is a major determinant of postoperative morbidity, functional decline, and survival in older adults with cancer, yet it is not consistently integrated into oncologic surgical decision-making. Comprehensive geriatric assessment (CGA) provides a multidimensional evaluation of physiological reserve, functional status, and patient priorities; but, its influence on treatment planning and survival in surgical oncology remains incompletely defined.

**Methods:**

We conducted a retrospective cohort study of adults aged ≥65 years referred for preoperative CGA between 2020 and 2023 at a dedicated geriatric surgery clinic within a large academic center. Patients were stratified by frailty status and CGA-derived clinical recommendation: Fit for Treatment, Prehabilitation & Surgery, or Other Intervention. Primary outcomes were modification of the initial treatment plan and overall survival. Associations were evaluated using multivariable logistic regression and Cox proportional hazards models.

**Results:**

Among 273 patients (median age 82 years; 42% female), treatment plans were modified in 21%, with modification rates increasing by frailty severity (non-frail 10%, mild–moderate 31%, severe 65%; p<0.001). Both frailty status and CGA recommendation independently predicted treatment-plan modification. Overall, 64% of patients underwent surgery, with marked variation by recommendation (Fit for Treatment 78%, Prehabilitation & Surgery 70%, Other Intervention 18%; p<0.001). Survival differed by frailty status (p=0.012) and CGA recommendation (p=0.008). In multivariable analysis, CGA recommendation was the strongest independent predictor of mortality, whereas frailty category was not independently associated with survival.

**Conclusion:**

CGA-derived recommendations substantially influenced surgical oncology treatment selection and were independently associated with survival, providing prognostic information beyond frailty status alone. Integrating CGA into preoperative surgical oncology workflows may support individualized, goal-concordant decision-making and help avoid potentially non-beneficial surgery in older adults with cancer.

## Introduction

Population ageing has led to a rapidly increasing number of older adults undergoing surgical treatment, including major oncologic procedures. Individuals aged ≥65 are projected to exceed 20% of the global population within two decades (1), while those aged ≥85 in the United States are expected rise from 2% in 2020 to 4.5% by 2050 (2). Adults aged ≥65 years already account for 35% of all outpatient surgeries, highlighting the need for age-adapted perioperative care.

Older surgical patients frequently present with increased medical complexity and diminished physiologic reserve translating into higher risks of postoperative morbidity, prolonged hospitalization, institutional discharge and mortality. Recovery following major surgery is often prolonged, and a substantial proportion of older adults experience persistent functional decline or reduced quality of life (3). Early identification of vulnerability through geriatric assessment has been shown to mitigate these risks and improve perioperative outcomes (4). Consequently, comprehensive and patient-centered perioperative planning has become increasingly important in surgical oncology, where treatment decisions must balance oncologic benefit against the potential of harm.

Conventional surgical risk assessment tools, including the American Society of Anesthesiologists Physical Status Classification System (ASA Score) (5), American College of Surgeons National Surgical Quality Improvement Program (ACS NSQIP) risk calculator (6), and Charlson Comorbidity Index (CCI) (7), primarily focus on comorbidities and procedure-related factors. While valuable, these tools have important limitations in older adults, as they inadequately capture geriatric-specific vulnerabilities such as frailty, cognitive impairment, and susceptibility to postoperative functional decline (8). As a result, they may underestimate perioperative risk and fail to predict patient-centered outcomes, including functional recovery and quality-of-life. In contrast, frailty assessment aims to quantify functional reserve, a key determinant of post-operative resilience in older adults (9).

A growing body of evidence demonstrates that frail older patients face a substantially increased risk of adverse surgical outcomes, including complications, institutionalization and death (10, 11). Screening strategies incorporating frailty assessment, together with targeted interventions such as prehabilitation, have been associated with improve surgical outcomes, including shorter hospital stays, reduced readmissions, and a higher likelihood of discharge to home (12). Comprehensive geriatric assessment (CGA), which systematically evaluates functional and cognitive status, nutritional health, medication burden, psychological well-being, and social support, enables identification of potentially modifiable vulnerabilities that may be addressed preoperatively to improve surgical tolerance and outcomes (13–15).

Recognizing the unique challenges of surgical care in older adults, a dedicated Geriatric Surgery Clinic was established in 2018 at Beilinson Hospital to provide structured preoperative CGA and multidisciplinary recommendations for patients being considered for major surgery.

The present study aimed to examine the clinical impact of CGA on surgical decision-making among older adults with gastrointestinal malignancies. Specifically, we evaluated whether CGA-based recommendations were associated with modification of the initial treatment plan and whether these recommendations and subsequent treatment decisions were associated with overall survival. We hypothesized that CGA-driven recommendations would meaningfully alter treatment planning and provide prognostic information beyond frailty status alone.

## Patients and methods

### Study design and patient population

We conducted a retrospective cohort study using a prospectively maintained database of consecutive patients who underwent Comprehensive Geriatric Assessment (CGA) between 2020 and 2023 at an outpatient Geriatric Surgery Clinic within a large academic referral center in Israel. Eligible participants were adults aged ≥65 years with a diagnosis of gastrointestinal malignancy who were considered candidates for major oncologic surgery and were referred for CGA with an initial treatment plan documented by the treating surgeon or oncologist.

The study was approved by the institutional review board (protocol 0154-19-RMC, approved on February 28, 2019). Patients were referred for geriatric evaluation after initial surgical or oncological assessment identified them as potential candidates for major surgery. Exclusion criteria included age <65 years, benign disease, or referral for CGA without surgical intent.

CGA was performed by a multidisciplinary team comprising a senior geriatrician, nurse, dietitian, physical therapist, and social worker. Assessment domains included medical comorbidity review, medication reconciliation, evaluation of functional status and mobility, nutritional assessment, cognitive screening, psychological health, and social support. CGA findings and recommendations were subsequently reviewed with the referring surgeon and/or discussed within a multidisciplinary Disease Management Team (DMT) meeting. Recommendations to postpone surgery for prehabilitation were generally made in patients with potentially reversible impairments, such as reduced mobility, functional dependence, malnutrition risk, polypharmacy, or suboptimal medical optimization. In contrast, transition to a non-operative approach was considered when severe frailty, advanced functional decline, major comorbidity burden, or limited physiological reserve suggested that surgical risk outweighed potential oncologic benefit. Importantly, these recommendations were discussed in a multidisciplinary team (MDT) meeting, and final decisions were reached by consensus rather than by the geriatrician alone.

### Outcome measures

The primary outcomes were (1) Treatment-plan modification and (2) Overall survival. Treatment-plan modification was defined as any change to the treating surgeon’s or oncologist’s initial management intent following CGA. Overall survival was, measured from the date of the first geriatric surgery clinic visit to death from any cause or last follow-up. Secondary outcomes were associations between frailty, geriatric assessment parameters, and likelihood of proceeding to surgery.

### Definition of geriatric clinic recommendations

Following CGA, each patient received one of three standardized recommendations:

1. Fit for Treatment: Proceed with planned surgery and/or oncologic treatment.
2. Prehabilitation & Surgery: Address modifiable vulnerabilities through targeted optimization prior to surgery, followed by reassessment for operative candidacy.
3. Other Intervention: Pursue alternatives to surgery, including non-operative oncologic management, watchful waiting, or palliative/supportive care.

### Definition of treatment plan modification

The initial treatment plan documented at referral was compared retrospectively with the treatment ultimately delivered. A *“Change in Treatment Plan”* was defined as any deviation from the original surgical or oncologic intent, including postponement of surgery, or transition to a non-operative approach.

### Data collection

Data were extracted from the prospective maintained database and included: Demographics (age, sex, marital status, education level); Clinical and laboratory variables (BMI, hemoglobin, albumin, and creatinine levels, and ASA classification); Geriatric assessment measures: frailty tools, functional and cognitive measures, mobility status, mood and anxiety scores; Oncologic and surgical/oncologic data (malignancy type, planned vs. received treatment, surgical procedure type, and one-year mortality).

### Frailty and geriatric assessment parameters

During the clinic’s first two years of the clinic’s operation, frailty was primarily assessed using the Clinical Frailty Scale score (CFS) (16). As the clinic matured, additional validated frailty tools were incorporated, including the FRAIL Scale, and the Frailty Index derived from Comprehensive Geriatric Assessment (FI-CGA) (4, 17). Patients were categorized by CFS classification whenever this measure was available. When CFS were unavailable, frailty classification was based on established FI-CGA thresholds.

CGA also incorporated additional validated geriatric domains: Cancer-related frailty risk was assessed using the G8 screening tool (18). The G8 screening tool was applied independently as a geriatric oncology screening instrument and was not considered a component of the CGA. Mobility status was categorized as independent, requiring partial assistance, or wheelchair dependent. Functional independence was evaluated using the Katz Index of Activities of Daily Living (19). Physical performance was assessed using the 4-Meter Gait Speed (20, 21); the 30-Second Sit-to-Stand Test (22); and Handgrip Strength (23). Cognitive screening was performed using Mini-Cog assessment (24). Symptoms of depression and anxiety were evaluated using the Patient Health Questionnaire-9 (PHQ-9) (25);and Generalized Anxiety Disorder-7 (GAD-7) (26).

As part of the multidisciplinary geriatric evaluation, patients were also assessed by a dietitian and a social worker. Nutritional evaluation included screening for malnutrition risk and dietary counseling when indicated. The social work assessment addressed social support, functional assistance at home, and discharge planning needs. When prehabilitation was recommended, it typically included individualized physiotherapy, nutritional optimization, and medical management of reversible deficits. However, detailed quantitative data regarding these components were not systematically collected for the purpose of this study.

### Frailty categorization thresholds

Frailty, functional status, cognitive function, and psychological measures were categorized using established and validated thresholds as follows:

* Clinical Frailty Scale (CFS): non-frail (≤4), mild–moderate frailty (5–6), severe frailty (≥7).
* FRAIL scale: robust =0, pre-frail = 1–2, frail ≥3;
* Frailty Index derived from CGA (FI-CGA): robust/pre-frail (<0.26), frail (0.26–0.40), severe frailty (>0.40).
* G8 screening tool: normal (>14), frailty risk (≤14).
* Katz Activities of Daily Living: independent (≥10), moderate assistance (6–9), severe impairment (≤5).
* Mini-Cog: unlikely impairment (≥3), cognitive impairment likely (≤2).
* Patient Health Questionnaire-9 (PHQ-9): minimal/mild (0–9), moderate (10–14), moderately severe (15–19), severe (≥20).
* Generalized Anxiety Disorder (GAD-)7: minimal (≤4), mild (5–9), moderate (10–14), severe (≥15).

### Statistical analysis

Analyses were structured according to the study objectives. Primary analyses evaluated factors associated with treatment-plan modification, while secondary analyses examined associations between frailty status, clinic recommendations, and overall survival.

Descriptive statistics are presented as n (%) for categorical variables and as mean ± SD or median [interquartile range, IQR] for continuous variables. Group comparisons for categorical variables were performed using Pearson’s χ² test or Fisher’s exact test when expected cell counts were small. Continuous variables were compared across groups using one-way analysis of variance (ANOVA) or the Kruskal–Wallis rank-sum test, as appropriate. The association between frailty category and clinic recommendation was assessed using χ² tests and summarized with Cramér’s V.

Time-to-event outcomes were analyzed with the Kaplan–Meier (KM) estimator, with between-group comparisons via log-rank tests. Medians survival times with 95% confidence intervals (CI) are reported; values were marked “NR” when not reached. To increase sensitivity to early differences when proportional hazards assumptions might not hold, the Gehan–Breslow test (ρ = 1) was prespecified. In such cases, 12-month KM estimates with Greenwood 95% CIs were also reported when appropriate. Median follow-up time was estimated using the reverse Kaplan-Meier method.

Cox proportional hazards models evaluated associations between frailty category, clinic recommendation, and overall survival. Multivariable models were adjusted for age, gender, Katz ADL category, mobility status, and G8 screening category. Nested likelihood-ratio tests evaluated the incremental contribution of clinic recommendation beyond frailty alone, the converse, and potential interaction between these predictors.

The binary endpoint of treatment plan modification (primary outcome) was analyzed using bias-reduced logistic regression, with odds ratios (ORs) and 95% confidence intervals (Cis) reported. Likelihood-ratio tests provided overall p-values, and marginal predicted probabilities were estimated for interpretability. All analyses were conducted in R version 4.3.0 (R Foundation for Statistical Computing, Vienna, Austria). Key packages included survival, ggsurvfit, gtsummary, brglm2, emmeans, ggplot2, ggpubr, and ggalluvial (27–32).

## Results

### Patient characteristics

A total of 273 older adults (median age 82 years [IQR 78–86]) were evaluated (**Table 1**). Increasing frailty severity was associated with significant differences in demographic and clinical characteristics. Severely frail patients were older than non-frail patients (median 84 vs 81 years; p = 0.006), more frequently widowed (52% vs 26%; p = 0.024), and had lower educational attainment (primary education 62% vs 21%; p = 0.001).

**Table 1:** Patient characteristics stratified by frailty category (clinical frailty scale, CFS).

Characteristic	Overall N = 273	Non-frail N = 167	Mild-moderate frailty N = 83	Severe frailty N = 23	P-value^1^
**Age, Median (IQR)**	82 (78 – 86)	81 (77 – 85)	84 (79 – 87)	84 (79 – 87)	**0.006**
Gender, n (%)					0.17
Female	115 (42)	63 (38)	40 (48)	12 (52)	
Male	158 (58)	104 (62)	43 (52)	11 (48)	
Marital Status, n (%)					0.024
Married	161 (59)	107 (64)	44 (53)	10 (43)	
Single	4 (1.5)	2 (1.2)	1 (1.2)	1 (4.3)	
Divorced	19 (7.0)	15 (9.0)	4 (4.8)	0 (0)	
Widow	89 (33)	43 (26)	34 (41)	12 (52)	
Education Level, n (%)					0.001
Primary	72 (27)	34 (21)	25 (30)	13 (62)	
Secondary	83 (31)	52 (32)	27 (33)	4 (19)	
Tertiary	112 (42)	78 (48)	30 (37)	4 (19)	
**BMI, Median (IQR)**	24.8 (22.0 – 27.9)	25.0 (22.1 – 28.1)	24.5 (22.0 – 27.8)	23.3 (21.2 – 27.4)	0.39
ASA Score, n (%)					<0.001
2	55 (32)	47 (42)	8 (16)	0 (0)	
3	93 (54)	54 (48)	30 (61)	9 (90)	
4	24 (14)	12 (11)	11 (22)	1 (10)	
**Hemoglobin (g/dL), Median (IQR)**	11.50 (10.20 – 12.70)	11.80 (10.30 – 12.70)	11.20 (9.80 – 12.50)	10.60 (9.65 – 11.50)	0.072
**Albumin (g/dL), Median (IQR)**	4.00 (3.60 – 4.30)	4.08 (3.60 – 4.30)	3.90 (3.40 – 4.20)	3.59 (3.40 – 3.80)	**0.004**
**Creatinine (mg/dL), Median (IQR)**	0.91 (0.73 – 1.16)	0.91 (0.73 – 1.12)	0.88 (0.70 – 1.24)	1.09 (0.70 – 1.38)	0.58
Malignancy Diagnosis, n (%)					0.52
Foregut	71 (26)	48 (29)	20 (24)	3 (13)	
Colorectal	119 (44)	68 (41)	39 (47)	12 (52)	
Hepato-Pancreato-Biliary/Other	83 (30)	51 (31)	24 (29)	8 (35)	
Treatment Recommendations After Geriatric Assessment, n (%)					<0.001
Fit for Treatment	97 (36)	79 (47)	14 (17)	4 (17)	
Prehabilitation & Surgery	132 (48)	81 (49)	47 (57)	4 (17)	
Other Intervention	44 (16)	7 (4.2)	22 (27)	15 (65)	
**Change in Medications^2^, n (%)**	147 (54)	76 (46)	58 (70)	13 (57)	**0.002**
**Social Assistance^2^, n (%)**	142 (52)	64 (38)	59 (71)	19 (83)	**<0.001**
**Surgery, n (%)**	176 (64)	124 (74)	44 (53)	8 (35)	**<0.001**
Surgery Type, n (%)					0.34
Foregut	45 (26)	34 (27)	11 (25)	0 (0)	
Colo-Rectal	84 (48)	55 (44)	24 (55)	5 (63)	
Hepato-Pancreato-Biliary/Other	47 (27)	35 (28)	9 (20)	3 (38)	
**Change in Treatment Plan, n (%)**	**58 (21)**	**17 (10)**	**26 (31)**	**15 (65)**	**<0.001**

Frailty categories were defined using the Clinical Frailty Scale (CFS): non-frail (CFS 1–3), mild-to-moderate frailty (CFS 4–6), and severe frailty (CFS ≥7).

^1^Kruskal-Wallis ^rank^ sum test; Pearson’s Chi-squared test; Fisher’s exact test.

^2^Recommendations following comprehensive geriatric assessment (CGA); BMI, body mass index; ASA Score, Anesthesiologists Physical Status Classification System.Bold values indicate a statistically significant p-value of <0.05.

Clinical characteristics also increased with frailty. ASA ≥3was present in 90% of severe frail compared with 59% of non-frail patients (p < 0.001). Albumin levels were significantly lower in severely frail patients (3.59 vs 4.08 g/dL; p = 0.004), while hemoglobin showed a similar trend (10.6 vs 11.8 g/d; p = 0.072). Distribution of gastrointestinal malignancy type did not differ significantly across frailty categories (p = 0.52).

### Geriatric clinic recommendations and treatment planning

CGA-derived recommendations closely aligned with frailty severity. Nearly half of non-frail patients (47%) were classified as Fit for Treatment, compared with 17% of severely frail patients (p < 0.001), while Other Intervention was recommended in 65% of severely frail versus 4% of non-frail patients.

Prehabilitation was advised in 48% overall, most frequently among patients with mild-to-moderately frailty (57%). Medication adjustments (54%) and social support referral (52%), increased significantly with frailty severity (p = 0.002 and p < 0.001, respectively).

Overall, 64% of patients proceeded to surgery, with rates declining across frailty groups (74% non-frail, 53% mild-to-moderate, 35% severe; p < 0.001).

**Figure 1:**
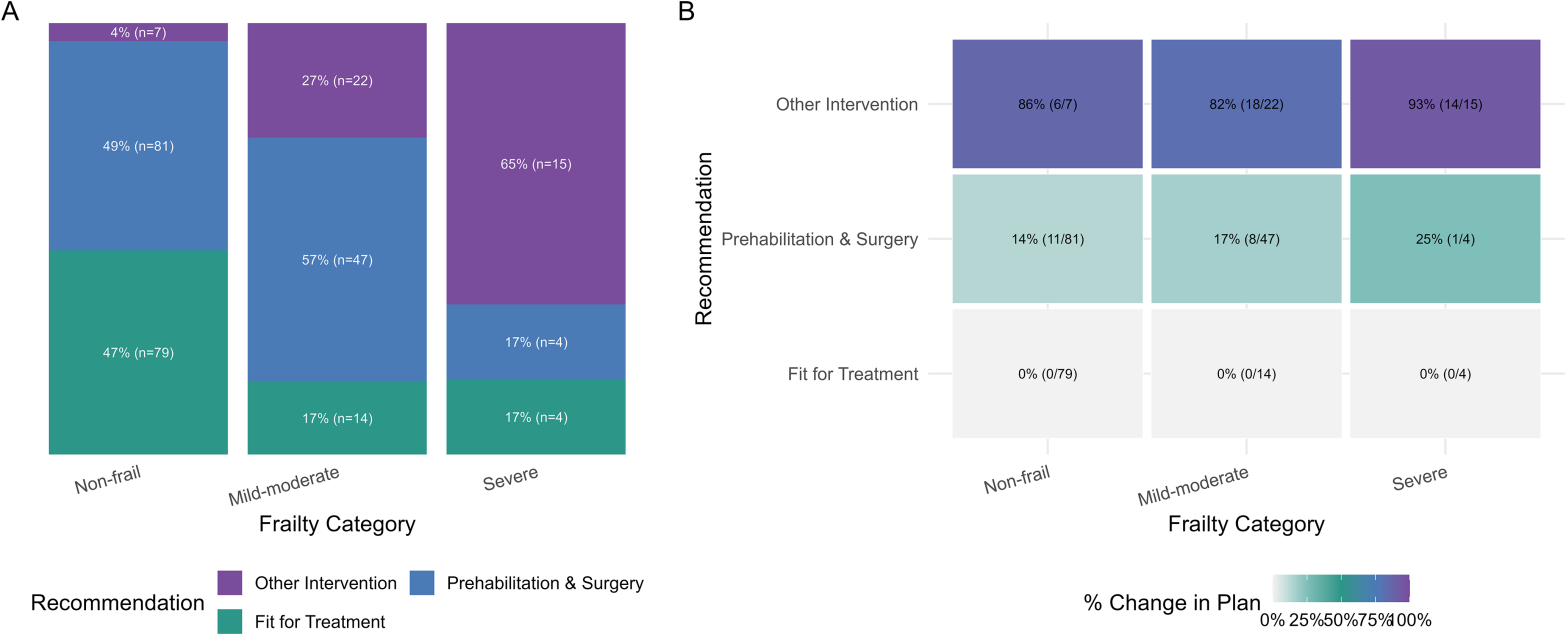
Association of frailty and clinical recommendations following CGA. **(A)** Stacked bars depict the proportion of clinical recommendations within each frailty category (labels: percent and sample size). *Frailty vs recommendation*: χ²(4) = 77.22, p = <0.001, Cramér’s V = 0.376). **(B)** Heatmap displays cross-tabulated frequencies of frailty and recommendation categories, color intensity proportional to the percentage of patients whose treatment plan changed. Logistic model for probability of change in treatment plan: effect of frailty p = <0.001, effect of clinical recommendation p = <0.001, interaction p = 1.000. Predicted probability of change in treatment plan for each clinical recommendation: Fit for Treatment 0.7%, Prehabilitation & Surgery 19.6%, Other Intervention 85.1%.

A modification of the initial treatment plan occurred in 21% of patients and increased markedly across frailty strata: 10% among non-frail patients, 31% among those with mild-to-moderate frailty, and 65% among patients with severe frailty (p < 0.001) (**Figure 1A**).

### Factors associated with change in treatment plan

Frailty status and CGA recommendations were both strongly associated with treatment-plan modification (**Table 2**). Among patients whose treatment plan changed, 45% had mild-to-moderate frailty and 26% had severe frailty, compared with 27% and 4%, respectively, among those without change (p < 0.001). No patients deemed Fit for Treatment experienced a change in treatment plan, compared with 34% of patients in the Prehabilitation & Surgery group and 66% in the Other Intervention group (p < 0.001).

**Table 2:** Characteristics associated with treatment plan modification.

Characteristic	Overall N = 273	No change in treatment plan N = 215	Change in treatment plan N = 58	P-value^1^
Clinical Frailty Scale, n (%)				<0.001
Non-frail	167 (61)	150 (70)	17 (29)	
Mild-moderate frailty	83 (30)	57 (27)	26 (45)	
Severe frailty	23 (8.4)	8 (3.7)	15 (26)	
Treatment Recommendations After Geriatric Assessment, n (%)				<0.001
Fit for Treatment	97 (36)	97 (45)	0 (0)	
Prehabilitation & Surgery	132 (48)	112 (52)	20 (34)	
Other Intervention	44 (16)	6 (2.8)	38 (66)	
**Surgery, n (%)**	176 (64)	159 (74)	17 (29)	**<0.001**
**1-Year Mortality, n (%)**	60 (22)	44 (20)	16 (28)	0.25

^1^Fisher’s exact test; Pearson’s Chi-squared test. Bold values indicate a statistically significant p-value of <0.05.

Surgery was performed in 74% of patients without plan modification versus 29% with modification (p < 0.001).

In bias-reduced logistic regression analyses, both frailty category and CGA- recommendation independently predicted treatment-plan modification, with additive rather than interactive effects (global p < 0.001 for each; no interaction) (**Figure 1B**), with the highest predicted probability among patients advised Other Intervention (85.1%), followed by Prehabilitation & Surgery (19.6%**),** and minimal among *Fit for Treatment* (0.7%).

### Survival outcomes

**Figure 2:**
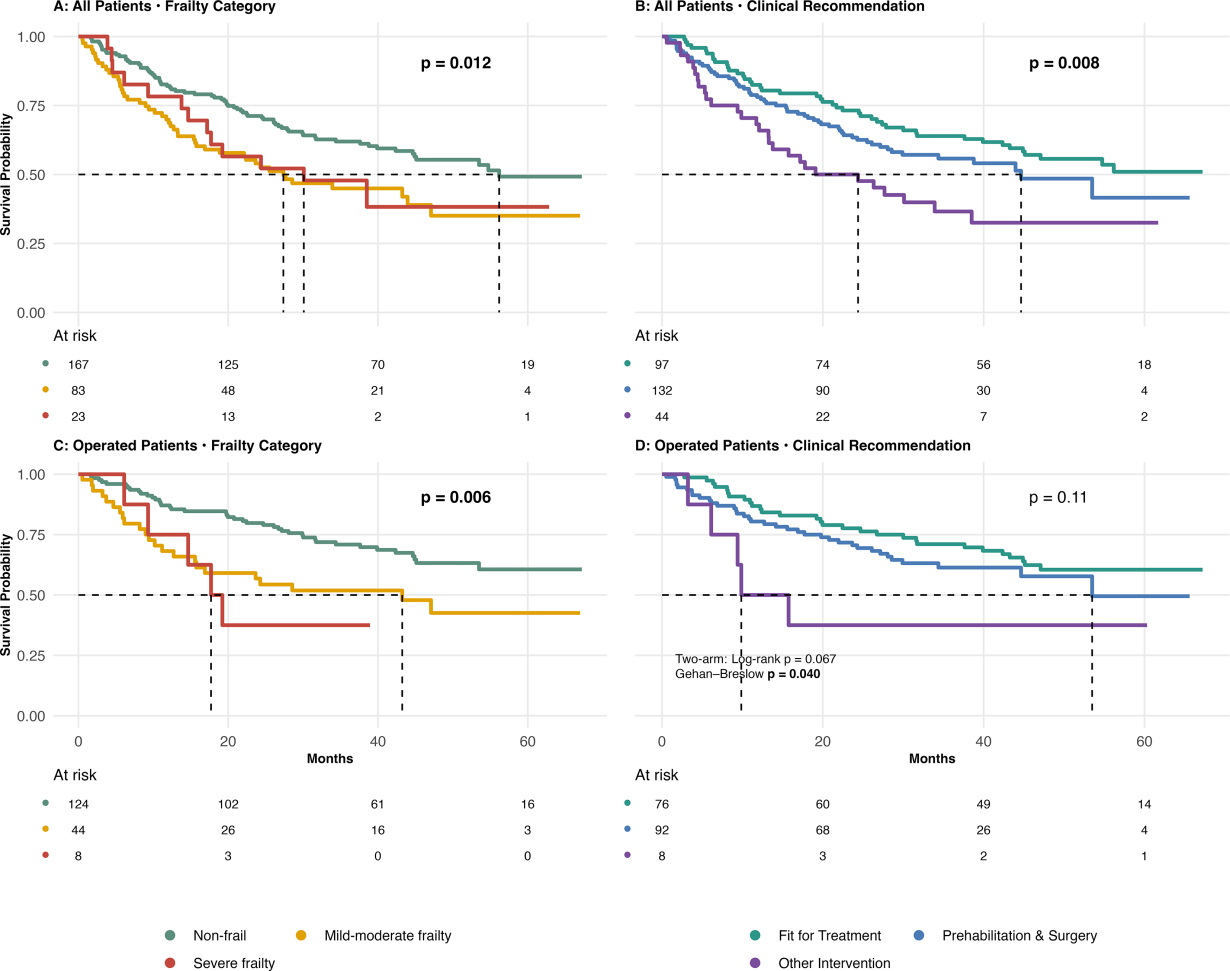
Kaplan–Meier plots of overall and postoperative survival by frailty status and clinical recommendation. Median follow-up was 44.9 months (95% CI 41.9–46.8), estimated using the reverse Kaplan–Meier method. **(A)** (All Patients, Frailty Category): Median survival - Non-frail: 56.2 months [44.9–NR]; Mild-moderate frailty: 27.4 months [16.9–NR]; Severe frailty: 30.1 months [17.2–NR] (p = 0.012). **(B)** (All Patients, Clinical Recommendation): Median survival - Fit for Treatment: NR; Prehabilitation & Surgery: 44.6 months [29.9–NR]; Other Intervention: 21.7 months [13.3–NR] (p = 0.008). **(C)** (Operated Patients, Frailty Category): Median survival - Non-frail: NR; Mild-moderate frailty: 43.2 months [15.7–NR]; Severe frailty: 18.5 months [14.7–NR] (p = 0.006). **(D)** (Operated Patients, Clinical Recommendation): Median survival - Fit for Treatment: NR; Prehabilitation & Surgery: 53.5 months [44.6–NR]; Other Intervention: 12.8 months [9.4–NR] (p = 0.11). *Among operated patients, 2-arm comparison (Fit for Treatment + Prehabilitation & Surgery vs Other Intervention)*: Log-rank p = 0.067 and Gehan–Breslow p = 0.040.; 12-months survival: 83.3% (95% CI 77.7%–89.0%) for Fit for Treatment + Prehabilitation & Surgery and 50.0% (95% CI 15.4%–84.6%) for Other Intervention (difference 33.3 percentage points; p = 0.063). NR, not reached.

After a median follow-up of 44.9 months (reverse KM 95% CI 41.9–46.8), overall survival differed significantly by frailty status and CGA recommendations (**Figure 2**). One-year mortality was higher among patients with treatment-plan change (28% vs 20%), though not statistically significant (p = 0.25). Survival declined with increasing frailty (log-rank p = 0.012). Median survival was 56.2 months in non-frail patients, 27.4 months in mild–to-moderate frailty. and 30.1 months in severe frailty. Survival was poorest among patients advised “Other Intervention” (p = 0.008). Median survival was not reached for “Fit for Treatment,” was 44.6 months) for “Prehabilitation & Surgery,” and 21.7 months for “Other Intervention”.

Among operated patients, frailty remained associated with survival (p = 0.006), whereas differences by recommendation demonstrated only a non-significant trend (p = 0.105).

In a two-group comparison of operated patients (“Fit for Treatment” plus “Prehabilitation & Surgery” vs. “Other Intervention”), the log-rank test demonstrated a borderline difference (p = 0.067), whereas the early-event–weighted (Gehan–Breslow p = 0.040), indicating an early survival disadvantage among patients recommended “Other Intervention.” Twelve-month survival estimates were 83.3% for the combined elective-surgery group and 50.0% for patients advised “Other Intervention” (p = 0.063).

In multivariable Cox models adjusting for demographic and geriatric variables, CGA recommendation independently predicted mortality, while frailty category did not. Compared “Fit for Treatment,” “Other Intervention” was associated with more than double the hazard of death (HR 2.27; p = 0.007), while those “Prehabilitation & Surgery” showed intermediate risk. (**Supplementary Figure S1**).

Model-comparison confirmed that adding clinic recommendation significantly improved a frailty-only model (p = 0.029), whereas frailty did not improve model performance (p = 0.84). Collectively, these findings indicate that CGA-derived recommendation provided stronger independent prognostic value than frailly status alone.

### Factors associated with treatment plan change

Bias-reduced logistic regression identify predictors of “Treatment Plan Change” (**Supplementary Table S1**). In univariable analyses, increasing frailty severity was strongly associated with treatment plan modification, with higher odds among patients with mild-to-moderate frailty (OR 4.66) and severe frailty (OR 17.0) compared with non-frail patients (global p < 0.001).

Clinic recommendations demonstrated the strongest association with treatment-plan change. Relative to “Fit for Treatment”, both “Other Intervention” (OR 634), and “Prehabilitation & Surgery” (OR 26.8), were linked to markedly increased odds of plan modification (global p < 0.001). G8 risk, Katz ADL impairment, and impaired mobility were also associated with plan change in univariable analyses (global p < 0.001).

In the multivariable model, clinic recommendations remained the dominant independent predictor. Compared with “Fit for Treatment”, “Other Intervention” (OR 945, 95% CI 50.0–17,849; p < 0.001) and “Prehabilitation & Surgery” (OR 38.9, 95% CI 3.02–501; p = 0.005) were strongly associated with treatment-plan modification. Partial mobility assistance also remained independently significant (OR 10.6, 95% CI 1.11–101; p = 0.040), while age demonstrated a modest inverse association (OR 0.91 per year, 95% CI 0.83–1.00; p = 0.049). After adjustment, CFS, G8, and Katz ADL were no longer independently predictive.

### Frailty and geriatric assessment measures

Measures of functional status, mobility, cognition, and psychological health demonstrated consistent and progressive impairment with increasing frailty severity are presented at a separated **Supplementary Table** (**Supplementary Table S2**).

### Summary of patient pathway

**Figure 3:**
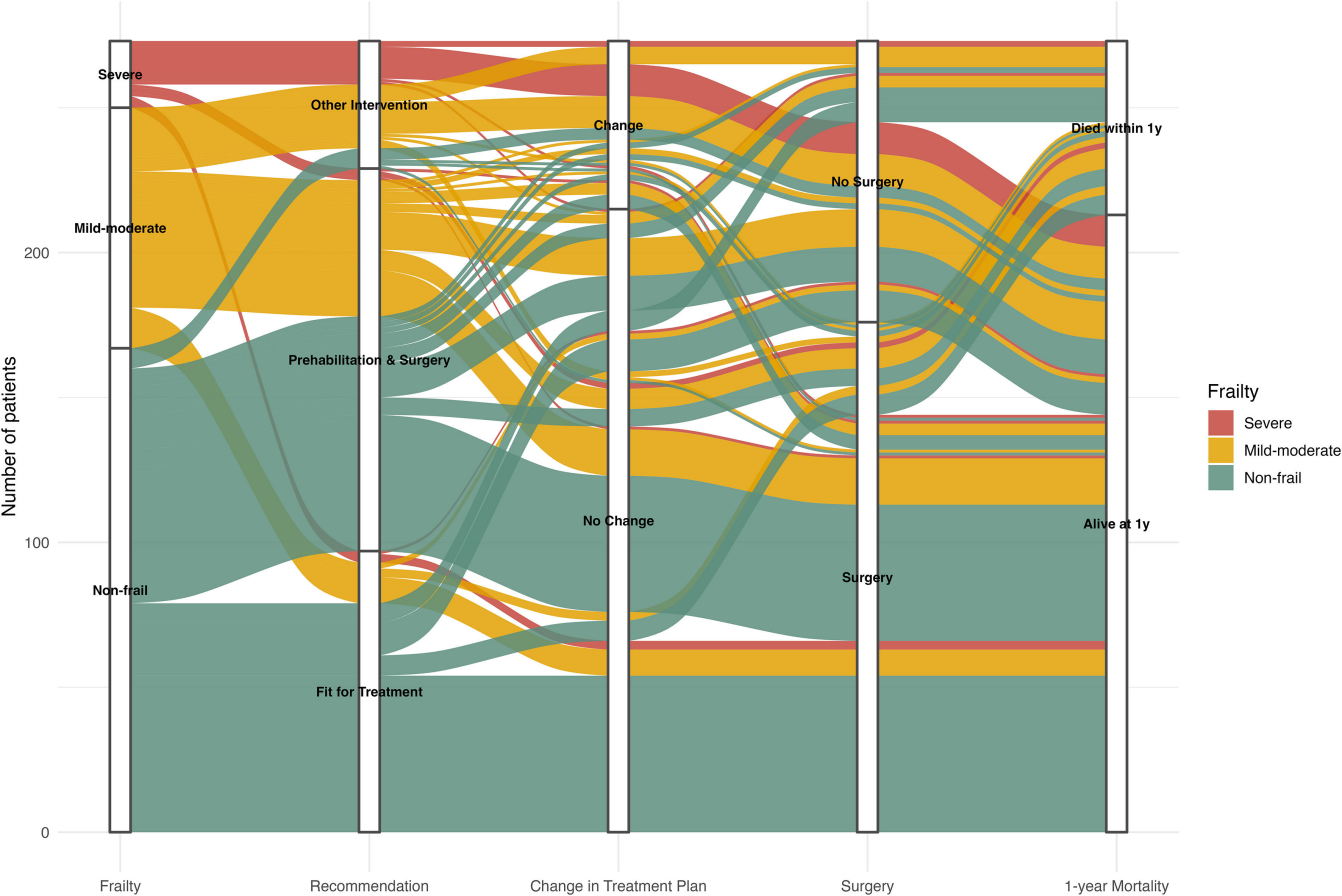
Alluvial diagram of patient pathways: frailty category → clinical recommendation → change in treatment plan → surgery → 1-year mortality. This image illustrates the sequential relationship between patients’ frailty classification, the geriatric clinic’s clinical recommendations following comprehensive geriatric assessment (CGA), subsequent change (or no change) in treatment plan, performance of surgery, and 1-year mortality outcomes. A strong association was observed between frailty category and clinical recommendation, between clinical recommendation and treatment-plan change, and between surgical intervention and plan modification (all p < 0.001). Overall, 21.2% (58/273) experienced a change in treatment plan, 64.5% (176/273) underwent surgery, and 22.0% (60/273) died within one year.

Patient trajectories linking frailty status, clinic recommendations, treatment-plan modification, surgery, and one-year mortality are illustrated in the alluvial diagram (**Figure 3**). A strong association was observed between frailty status and clinic recommendation (χ²(4) = 66.9; p < 0.001; Cramér’s V = 0.39).

Overall, 21% (58/273) of patients underwent modification of the initial treatment plan. The rate of plan change increased markedly with frailty severity: 10% in non-frail patients, 31% in mild–to-moderate frailty, and 65% (15/23) in severe frailty (p < 0.001);. Treatment plan modification also differed substantially by clinic recommendation (p < 0.001): occurring in 0% of patients deemed “Fit for Treatment”, 20% of those recommended “Prehabilitation & Surgery,” and 86% of those advised “Other Intervention”.

Surgery was performed more frequently when the treatment-plan remained unchanged than modified (74.0%, vs 29.3% p < 0.001). Overall, one-year mortality for the cohort was 22.0%.

## Discussion

In this single-center cohort of older adults referred for preoperative comprehensive geriatric assessment (CGA) prior to consideration of major surgery for gastrointestinal malignancies, we found that treatment plans were modified in one in five patients with the likelihood of modification increasing in parallel with frailty severity. Beyond frailty status alone, CGA-derived clinical recommendation emerged as the strongest determinant of subsequent treatment plan modification and downstream surgical decision-making. Importantly, these recommendations were also independently associated with overall survival, exceeding the prognostic value of frailty measures when adjusted for functional status, mobility, and geriatric screening tools.

Frailty, reflecting diminished physiological reserve and vulnerability to stressors, is a well-established predictor of postoperative morbidity and mortality in older surgical oncology patients (33, 34). In our cohort, increasing frailty severity was associated with both higher rates of treatment-plan modification and worse overall survival. CGA provides a multidisciplinary evaluation that extends beyond frailty to encompass functional capacity, cognition, nutrition, mobility, psychological health, and social support (35). Multiple professional societies have recommend routine incorporation of geriatric assessment in the care of older adults with cancer (36–39). Evidence demonstrates that formal CGA outperforms screening tools and clinician judgment in identifying frailty and predicting outcomes (40, 41). In our study, the G8 screening tool demonstrated limited discriminative value. Despite labeling 83% of the cohort as “frailty risk” by the G8 score, only 20% of these patients ultimately received an “Other Intervention” recommendation, and surgical rates were similar across G8 groups. In multivariable analysis, the G8 did not independently predict treatment-plan modification. These findings align with concerns reported in other surgical oncology settings that the G8 alone may insufficiently differentiate risk in older adults with gastrointestinal malignancies (42).

Although the G8 has been widely adopted as a rapid screening tool to identify older cancer patients who may benefit from comprehensive geriatric assessment, its specificity in surgical populations may be limited. Russo et al. found that while the G8 demonstrated good sensitivity for detecting vulnerability compared with comprehensive geriatric assessment, its ability to discriminate patients requiring treatment modification was less robust than more comprehensive evaluation approaches (43).

Among severely frail patients, the geriatric clinic team recommended alternatives management options such as palliative care, supportive oncological treatment when feasible or non-intervention, in 65% of cases, closely paralleling the observed rate of treatment-plan modification. Importantly, severe frailty did not reflect terminal status (CFS 9), and surgical candidacy in these cases was determined through multidisciplinary evaluation considering overall risk–benefit balance. Increasing frailty severity was strongly associated with a higher likelihood of changes in treatment plan. The gradient in plan change, ranging from 10% in non-frail patients to 65% in those with severe frailty, underscores frailty’s influence on perioperative risk, vulnerability to postoperative decline, and the potential for non-beneficial or harmful operations. Early identification of frailty through CGA enables clinicians to redirect care toward approaches better aligned with patient priorities, thereby optimizing outcomes and reducing treatment burden.

In our study, CGA-derived clinical recommendations played a pivotal role in shaping patient trajectories. influencing both decision-making and patient choice of the proposed intervention. Patients deemed “Fit for Treatment” rarely deviated from their initial surgical or oncologic plan, whereas those categorized as “Other Intervention” infrequently proceeded to surgery. In regression analyses, the recommendation category was the strongest predictor of treatment-plan modification—exceeding the explanatory power of frailty alone. These findings illustrate that CGA recommendations synthesize frailty, function, comorbidity, social support, and patient preference into an integrated clinical judgment that informs both surgeon and patient decision-making. Prior work highlights the value of structured, interdisciplinary discussions in achieving patient-centered, goal-concordant care, and our results reinforce this principle (44).

The “Prehabilitation & Surgery” group comprised approximately 40% of the cohort and largely represented patients with mild-to-moderate frailty. Only 15% of experienced a treatment-plan change, and 70% ultimately underwent surgery, rates approaching those of the “Fit for Treatment” group. As prehabilitation recommendations were reached through multidisciplinary consensus and often reflected short-term optimization rather than alteration of the surgical intent, the observed rate of formal treatment-plan modification should not be interpreted as discordance between geriatric and surgical decision-making. Their median overall survival (44.6 months) was substantially higher than that of the “Other Intervention” group (21.7 months) and closer to that of the fittest patients. These findings are consisted with growing evidence supporting targeted prehabilitation and multidisciplinary optimization to improve outcomes in frail or marginal-risk surgical candidates (45–48).

Importantly, survival analyses demonstrated that while both frailty and clinical recommendations were associated with mortality, only the recommendation category remained independently predictive after adjustment for frailty, mobility, functional dependence, and G8 risk. Model-comparison testing showed that adding clinical recommendations significantly improved prognostic accuracy, whereas adding frailty did not This suggests that the CGA recommendation functions as a clinically meaningful synthesis of physiologic reserve, functional capability, comorbidity, and anticipated treatment tolerance, offering a pragmatic tool for risk stratification and shared decision-making in surgical oncology.

Taken together, these results reinforce the value of CGA not merely as a frailty detection tool, but as a structured mechanism for integrating multidimensional patient information into actionable, patient-centered recommendations. The geriatric team’s assessment, combined with collaborative discussions involving patients and surgeons, supports decision-making that avoids potentially futile operations, identifies candidates most likely to benefit from prehabilitation, and aligns treatment plans with individual goals and functional priorities.

This study has several limitations. Its retrospective, single-center design may limit generalizability, and referral bias is inherent, as only patients deemed potential surgical candidates were evaluated. Frailty assessment tools evolved over the study period, reflecting real-world clinical practice but introducing heterogeneity; however, CGA recommendations were generated through a consistent multidisciplinary process. Detailed oncologic staging data were not uniformly available, and postoperative functional outcomes and quality-of-life measures were not assessed. Detailed data regarding nutritional and social work assessments, specific prehabilitation components, and objective response to these interventions were not systematically collected, limiting our ability to evaluate their independent contribution to outcomes. We did not systematically evaluate objective changes following CGA-guided interventions, limiting conclusions regarding the effectiveness of prehabilitation. Cause-specific mortality was not available, limiting our ability to distinguish between cancer-related, treatment-related, and competing-cause mortality. Therefore, conclusions regarding the appropriateness of surgical omission based on survival outcomes should be interpreted with caution. Despite these limitations, the study provides pragmatic, real-world evidence of how CGA can meaningfully influence surgical oncology decision-making.

In summary, CGA-derived recommendations strongly influenced treatment planning and were independently associated with survival among older adults evaluated for major oncologic surgery. These findings reinforce existing literature supporting CGA as a cornerstone of geriatric oncology care and support integrating CGA into routine preoperative workflows to promote individualized, goal-concordant decisions, identify patients most likely to benefit from prehabilitation, and avoid potentially non-beneficial surgery. Future prospective studies should evaluate standardized CGA referral pathways, structured prehabilitation programs, and patient-centered outcomes, including functional recovery and quality of life, to further refine best practices for geriatric surgical oncology.

## Data Availability

The raw data supporting the conclusions of this article will be made available by the authors, without undue reservation.

## References

[1] World Health Organization . Ageing and health. Available online at: https://www.who.int/news-room/fact-sheets/detail/ageing-and-health (Accessed January 2, 2026).

[2] U.S. Census Bureau . An aging nation: projected number of children and older adults. Available online at: https://www.census.gov/library/visualizations/2018/comm/historic-first.html (Accessed January 2, 2026).

[3] Cooper L Abbett SK Feng A Morris M Partridge JS Chow WB . Launching a geriatric surgery center: recommendations from the Society for Perioperative Assessment and Quality Improvement. J Am Geriatr Soc. (2020) 68(9):2134–42. doi: 10.1111/jgs.16681. PMID: 32662064

[4] Cooper L Loewenthal J Frain LN Hornor MA Ko CY Mohanty S . From research to bedside: incorporation of a CGA-based frailty index among multiple comanagement services. J Am Geriatr Soc. (2022) 70:90–8. doi: 10.1111/jgs.17446. PMID: 34519037 PMC9056009

[5] Mayhew D Mendonca V Murthy BVS . A review of ASA physical status – historical perspectives and modern developments. Anesthesia. (2019) 74:373–9. doi: 10.1111/anae.14569. PMID: 30648259

[6] Ingraham AM Richards KE Hall BL Ko CY . Quality improvement in surgery: The American College of Surgeons National Surgical Quality Improvement Program approach. Adv Surg. (2010) 44:251–67. doi: 10.1016/j.yasu.2010.05.003. PMID: 20919525

[7] Charlson ME Pompei P Ales KL MacKenzie CR . A new method of classifying prognostic comorbidity in longitudinal studies: development and validation. J Chronic Dis. (1987) 40:373–83. doi: 10.1016/0021-9681(87)90171-8. PMID: 3558716

[8] Grudzinski AL Aucoin S Talarico R Moloo H Lalu MM McIsaac DI . Comparing the predictive accuracy of frailty instruments applied to preoperative electronic health data for adults undergoing noncardiac surgery. Br J Anaesth. (2022) 129:506–14. doi: 10.1016/j.bja.2022.07.019. PMID: 36031416

[9] Khan KT Hemati K Donovan AL . Geriatric physiology and the frailty syndrome. Anesthesiol Clin. (2019) 37:453–74. doi: 10.1016/j.anclin.2019.04.006. PMID: 31337478

[10] Tan KY Kawamura YJ Tokomitsu A Tang T . Assessment for frailty is useful for predicting morbidity in elderly patients undergoing colorectal cancer resection whose comorbidities are already optimized. Am J Surg. (2012) 204:139–43. doi: 10.1016/j.amjsurg.2011.08.012. PMID: 22178483

[11] Gong S Qian D Riazi S McIsaac DI Wijeysundera DN Karkouti K . Association between the FRAIL scale and postoperative complications in older surgical patients: a systematic review and meta-analysis. Anesth Analg. (2023) 136:251–61. doi: 10.1213/ANE.0000000000006272. PMID: 36638509 PMC9812423

[12] Paille M Senage T Roussel JC Lelong B Hacot JP Leclercq C . Association of preoperative geriatric assessment with length of stay after combined cardiac surgery. Ann Thorac Surg. (2021) 112:763–9. doi: 10.1016/j.athoracsur.2020.09.041. PMID: 33227273

[13] Mohile SG Mohamed MR Xu H Culakova E Loh KP Magnuson A . Evaluation of geriatric assessment and management on the toxic effects of cancer treatment (GAP70+): a cluster-randomized study. Lancet. (2021) 398:1894–904. doi: 10.1016/S0140-6736(21)01789-X. PMID: 34741815 PMC8647163

[14] Parker SG Mccue P Phelps K McCleod A Arora S Nockels K . What is comprehensive geriatric assessment (CGA)? An umbrella review. Age Ageing. (2018) 47:149–55. doi: 10.1093/ageing/afx166. PMID: 29206906

[15] Ellis G Gardner M Tsiachristas A Langhorne P Burke O Harwood RH . Comprehensive geriatric assessment for older adults admitted to hospital. Cochrane Database Syst Rev. (2017) 2017. doi: 10.1002/14651858.CD006211.pub3. PMID: 28898390 PMC6484374

[16] Church S Rogers E Rockwood K Theou O . A scoping review of the Clinical Frailty Scale. BMC Geriatr. (2020) 20. doi: 10.1186/s12877-020-01801-7. PMID: 33028215 PMC7540438

[17] Kojima G Iliffe S Walters K . Frailty index as a predictor of mortality: a systematic review and meta-analysis. Age Ageing. (2018) 47:193–200. doi: 10.1093/ageing/afx162. PMID: 29040347

[18] Depoorter V Vanschoenbeek K Decoster L . Long-term health-care utilization in older patients with cancer and the association with the Geriatric 8 screening tool. Lancet Healthy Longev. (2023) 4:e326–36. doi: 10.1016/S2666-7568(23)00081-8. PMID: 37327806

[19] Katz S Ford AB Moskowitz RW Jackson BA Jaffe MW . Studies of illness in the aged: the index of ADL. JAMA. (1963) 185(12):914–9. doi: 10.1001/jama.1963.03060120024016. PMID: 14044222

[20] Deeb AL Garrity M Cooper L Chow WB Partridge JS Feng A . Implementing 4-meter gait speed as a routine vital sign in a thoracic surgery clinic. J Geriatr Oncol. (2023) 14. doi: 10.1016/j.jgo.2023.101481. PMID: 37060720 PMC10445274

[21] Singh A Xie Y Mazzola E Velotta JB Yang CJ D’Amico TA . Gait speed as a measure of frailty and outcomes after lung resection. Ann Surg Oncol. (2025) 32:4181–8. doi: 10.1245/s10434-025-17066-6. PMID: 40016615 PMC12857551

[22] Lein DH Alotaibi M Almutairi M Singh H . Normative reference values and validity for the 30-Second Chair-Stand Test in healthy young adults. Int J Sports Phys Ther. (2022) 17:907–14. doi: 10.26603/001c.36432. PMID: 35949374 PMC9340829

[23] Mehmet H Yang AWH Robinson SR . Measurement of hand grip strength in the elderly: A scoping review with recommendations. J Bodyw Mov Ther. (2020) 24:235–43. doi: 10.1016/J.JBMT.2019.05.029. PMID: 31987550

[24] Sherman JB Chatterjee A Urman RD Moucha CS Sinha AC Gibbons MM . Implementation of routine cognitive screening in the preoperative assessment clinic. A A Pract. (2019) 12:125–7. doi: 10.1213/XAA.0000000000000891. PMID: 30234511

[25] Negeri ZF Levis B Sun Y He C Krishnan A Wu Y . Accuracy of the PHQ-9 for screening to detect major depression: updated systematic review and IPD meta-analysis. BMJ. (2021) 375. doi: 10.1136/bmj.n2183. PMID: 34610915 PMC8491108

[26] Saracino RM Kehoe LA Sohn MB Applebaum AJ Lichtenthal WG Breitbart W . Psychometric properties of the GAD-7 in older adults with advanced cancer. Psychooncology. (2024) 33. doi: 10.1002/pon.70012. PMID: 39482282 PMC11905212

[27] Therneau TM . Survival: Survival Analysis (2024). Available online at: https://github.com/therneau/survival (Accessed January 2, 2026).

[28] Kosmidis I Pagui ECK . Brglm2: Bias Reduction in Generalized Linear Models (2025). Available online at: https://github.com/ikosmidis/brglm2 (Accessed January 2, 2026).

[29] Lenth RV . Emmeans: Estimated Marginal Means, Aka Least-Squares Means (2025). Available online at: https://rvlenth.github.io/emmeans/ (Accessed January 2, 2026).

[30] Sjoberg DD Baillie M Fruechtenicht C Haesendonckx S Treis T . Ggsurvfit: Flexible Time-to-Event Figures 2 (025). Available online at: https://github.com/pharmaverse/ggsurvfit (Accessed January 2, 2026).

[31] Sjoberg DD Whiting K Curry M Lavery JA Larmarange J . Reproducible Summary tables with the gtsummary package. R J. (2021) 13:570–80. doi: 10.32614/RJ-2021-053. PMID: 41351832

[32] Wickham H Chang W Henry L . Ggplot2: create elegant Data Visualisations Using the Grammar of Graphics (2025). Available online at: https://ggplot2.tidyverse.org.

[33] Ommundsen N Wyller TB Nesbakken A Jordhøy MS Bakka AO Skovlund E . Frailty is an Independent predictor of survival in older patients with colorectal cancer. Oncologist. (2014) 19:1268–75. doi: 10.1634/theoncologist.2014-0237. PMID: 25355846 PMC4257747

[34] Huisman MG Audisio RA Ugolini G Montroni I Vigano A Spiliotis J . Screening for predictors of adverse outcome in onco-geriatric surgical patients. Eur J Surg Oncol. (2015) 41:844–51. doi: 10.1016/j.ejso.2015.02.018. PMID: 25935371

[35] Zietlow KE Wong S Heflin MT McDonald SR Colón-Emeric CS Hastings SN . Geriatric preoperative optimization: a Review. Am J Med. (2022) 135:39–48. doi: 10.1016/j.amjmed.2021.07.028. PMID: 34416164 PMC8688225

[36] Loh KP Liposits G Arora SP Williams GR Wildiers H Lichtman SM . The role of geriatric assessment and management in older adults with cancer: ESMO/SIOG position paper. ESMO Open. (2024) 9:103657. doi: 10.1016/j.esmoop.2024.103657. PMID: 39232585 PMC11410714

[37] Ma M Zhang L Rosenthal R Finlayson E Russell MM . The ACS Geriatric Verification Program and the practicing colorectal surgeon. Semin Colon Rectal Surg. (2020) 31. doi: 10.1016/j.scrs.2020.100779. PMID: 33041604 PMC7531280

[38] Dotan E Walter LC Browner IS Mohile SG Hurria A Owusu C . Older adult oncology, version 1.2021: updates to the NCCN guidelines. J Natl Compr Canc Netw. (2021) 19:1006–19. doi: 10.6004/jnccn.2021.0043. PMID: 34551388

[39] Saur NM Davis BR Montroni I Shahrokni A Rostoft S O’Connor ES . ASCRS clinical practice guidelines for perioperative evaluation and management of frailty among older adults undergoing colorectal surgery. Dis Colon Rectum. (2022) 65:473–88. doi: 10.1097/DCR.0000000000002410. PMID: 35001046

[40] Kirkhus L Benth JŠ Rostoft S Grønberg BH Hjermstad MJ Selbæk G . Geriatric assessment is superior to clinical judgement in identifying frailty. Br J Cancer. (2017) 117:470–7. doi: 10.1038/bjc.2017.202. PMID: 28664916 PMC5558687

[41] Kirkhus L Šaltytė Benth J Grønberg BH Hjermstad MJ Selbæk G Jordhøy MS . Frailty identified by geriatric assessment is associated with poor functioning, high symptom burden and increased risk of physical decline in older cancer patients: Prospective observational study. Palliat Med. (2019) 33:312–22. doi: 10.1177/0269216319825972. PMID: 30712456 PMC6376598

[42] Bruijnen CP Heijmer A van Harten-Krouwel DG van den Bos F de Bree R van der Velde N . Validation of the G8 screening tool in older patients with cancer considered for surgical treatment. J Geriatr Oncol. (2021) 12:793–8. doi: 10.1016/j.jgo.2020.10.017. PMID: 33172806

[43] Russo C Giannotti C Signori A Basso U Bongiovanni A Tucci M . Predictive values of two frailty screening tools in older patients with solid cancer: a comparison of SAOP2 and G8. Oncotarget. (2018) 9:35056–68. doi: 10.18632/oncotarget.26147. PMID: 30416679 PMC6205549

[44] Van der Wal-Huisman H van Leeuwen BL Stiggelbout AM Hiligsmann M van den Bos F de Bock GH . Integrated oncological treatment decision-making: creating a practice of patient-centered decision-making. Patient Educ Couns. (2025) 131. doi: 10.1016/j.pec.2024.108555. PMID: 39579519

[45] Dezube AR Cooper L Jaklitsch MT . Prehabilitation of the thoracic surgery patient. Thorac Surg Clin. (2020) 30:249–58. doi: 10.1016/j.thorsurg.2020.04.004. PMID: 32593358

[46] Sabajo CR ten Cate DWG Heijmans MHM van der Meij E van der Sluis PC Slooter GD . Prehabilitation in colorectal cancer surgery improves outcome and reduces hospital costs. Eur J Surg Oncol. (2024) 50. doi: 10.1016/j.ejso.2023.107302. PMID: 38043359

[47] Molenaar CJL Minnella EM Coca-Martinez M Awasthi R Carli F Scheede-Bergdahl C . Effect of multimodal prehabilitation on reducing postoperative complications and enhancing functional capacity following colorectal cancer surgery: the PREHAB randomized clinical trial. JAMA Surg. (2023) 158:572–81. 36988937 10.1001/jamasurg.2023.0198 PMC10061316

[48] Heil TC Verdaasdonk EGG Maas HAAM van der Hulst RRWJ van der Meij E Slooter GD . Improved postoperative outcomes after prehabilitation for colorectal cancer surgery in older patients: an emulated Target trial. Ann Surg Oncol. (2023) 30:244–54. doi: 10.1245/s10434-022-12623-9. PMID: 36197561 PMC9533971

