# Supplementary material for: From frailty to decision: impact of comprehensive geriatric assessment on surgical planning and outcomes in older adults with gastrointestinal malignancies
Source: Front Oncol. 2026 Apr 13;16:1788002. doi: 10.3389/fonc.2026.1788002 (PMC13111140; doi:10.3389/fonc.2026.1788002)
Supplement: Supplementary file 1 [file SupplementaryFile1.docx]

***From frailty to decision: impact of comprehensive geriatric assessment on surgical***

***planning and outcomes in older adults with gastrointestinal malignancies***

**SUPPLEMENTAL MATERIAL**

*Legend*

- Supplementary Table S1. Frailty and geriatric assessment measures
- Supplementary Table S2. Logistic regression for “Change in Treatment Plan”
- Supplementary Figure S1. Adjusted hazard ratios for overall survival.

**Supplementary Table S1. Frailty and geriatric assessment measures**

| **Characteristic** | **Overall** N = 273 | **Non-frail** N = 167 | **Mild-moderate frailty** N = 83 | **Severe frailty** N = 23 | **p-value***^1^* |
| --- | --- | --- | --- | --- | --- |
| **G8 Score, n (%)** |  |  |  |  | **<0.001** |
| Normal | 44 (17) | 40 (25) | 3 (3.9) | 1 (5.6) |  |
| Frailty risk | 209 (83) | 119 (75) | 73 (96) | 17 (94) |  |
| **Mobility, n (%)** |  |  |  |  | **<0.001** |
| Independent | 223 (82) | 163 (98) | 55 (67) | 5 (22) |  |
| Partial assistance | 38 (14) | 3 (1.8) | 24 (29) | 11 (48) |  |
| Complete assistance | 11 (4.0) | 1 (0.6) | 3 (3.7) | 7 (30) |  |
| **Katz Index of Independence in Activities of Daily Living, n (%)** |  |  |  |  | **<0.001** |
| Independent | 219 (81) | 165 (99) | 52 (64) | 2 (8.7) |  |
| Moderate assistance | 32 (12) | 1 (0.6) | 24 (30) | 7 (30) |  |
| Severe impairment | 20 (7.4) | 1 (0.6) | 5 (6.2) | 14 (61) |  |
| **4-Meter Walk Time (s), Median (IQR)** | 4.0 (3.0 – 6.0) | 4.0 (3.0 – 4.0) | 5.5 (4.0 – 8.0) | 0.0 (0.0 – 5.0) | **<0.001** |
| **30-Second Sit-to-Stand Test (repetitions), Median (IQR)** | 9.0 (0.5 – 12.0) | 11.0 (9.0 – 13.0) | 5.0 (0.0 – 8.5) | 0.0 (0.0 – 4.0) | **<0.001** |
| **Hand Grip Strength (kg), Median (IQR)** | 25 (16 – 35) | 28 (20 – 40) | 22 (14 – 30) | 14 (8 – 18) | **<0.001** |
| **Mini-Cog Score, n (%)** |  |  |  |  | **<0.001** |
| Unlikely impairment | 107 (66) | 71 (85) | 34 (53) | 2 (14) |  |
| Cognitive impairment likely | 55 (34) | 13 (15) | 30 (47) | 12 (86) |  |
| **Patient Health Questionnaire-9 (PHQ-9), n (%)** |  |  |  |  | **<0.001** |
| Minimal/Mild | 99 (63) | 64 (77) | 31 (53) | 4 (29) |  |
| Moderate | 28 (18) | 13 (16) | 14 (24) | 1 (7.1) |  |
| Moderately severe | 21 (13) | 6 (7.2) | 9 (15) | 6 (43) |  |
| Severe | 8 (5.1) | 0 (0) | 5 (8.5) | 3 (21) |  |
| **Generalized Anxiety Disorder-7 (GAD-7), n (%)** |  |  |  |  | **0.036** |
|  |  |  |  |  |  |
| Minimal | 78 (50) | 51 (61) | 23 (39) | 4 (29) |  |
| Mild | 44 (28) | 20 (24) | 20 (34) | 4 (29) |  |
| Moderate | 21 (13) | 8 (9.6) | 10 (17) | 3 (21) |  |
| Severe | 13 (8.3) | 4 (4.8) | 6 (10) | 3 (21) |  |
| *^1^*Fisher's exact test; Kruskal-Wallis rank sum test | | | | | |

**Supplementary Table S2. Logistic regression for “Change in Treatment Plan”**

|  | **Univariable** | | | | **Multivariable** | |
| --- | --- | --- | --- | --- | --- | --- |
| **Characteristic** | **N** | **OR** **(95% CI)** | **p-value** | **Global p (Wald)** | **OR** **(95% CI)** | **p-value** |
| **Age (years)** | 214 | 1.04 (0.98 to 1.10) | 0.189 | 0.2 | 0.93 (0.85 to 1.01) | 0.094 |
| **Gender** | 214 |  |  | 0.11 |  |  |
| Female |  | — |  |  | — |  |
| Male |  | 0.58 (0.29 to 1.14) | 0.114 |  | 1.08 (0.35 to 3.32) | 0.888 |
| **Body Mass Index (BMI, kg/m²)** | 214 | 0.96 (0.89 to 1.04) | 0.306 | 0.3 | 1.08 (0.97 to 1.21) | 0.145 |
| **Hemoglobin (g/dL)** | 214 | 0.90 (0.75 to 1.08) | 0.265 | 0.3 | 0.88 (0.67 to 1.16) | 0.371 |
| **Clinical Frailty Scale** | 214 |  |  | **<0.001** |  |  |
| Non-frail |  | — |  |  | — |  |
| Mild-moderate frailty |  | 4.67 (2.15 to 10.1) | **<0.001** |  | 0.71 (0.21 to 2.46) | 0.591 |
| Severe frailty |  | 19.9 (5.47 to 72.6) | **<0.001** |  | 1.90 (0.14 to 26.2) | 0.630 |
| **Recommendation** | 214 |  |  | **<0.001** |  |  |
| Fit for Treatment |  | — |  |  | — |  |
| Other Intervention |  | 747 (39.1 to 14,271) | **<0.001** |  | 914 (51.7 to 16,139) | **<0.001** |
| Prehabilitation & Surgery |  | 28.3 (1.64 to 488) | **0.021** |  | 39.8 (3.08 to 514) | **0.005** |
| **G8 Score** | 214 |  |  | **0.018** |  |  |
| Normal |  | — |  |  | — |  |
| Frailty risk |  | 7.66 (1.41 to 41.5) | **0.018** |  | 8.04 (0.93 to 69.8) | 0.059 |
| **Katz ADL** | 214 |  |  | **<0.001** |  |  |
| Independent |  | — |  |  | — |  |
| Moderate assistance |  | 8.62 (3.39 to 21.9) | **<0.001** |  | 1.65 (0.21 to 13.3) | 0.635 |
| Severe impairment |  | 7.92 (2.24 to 28.0) | **0.001** |  | 0.07 (0.00 to 2.05) | 0.122 |
| **Mobility** | 214 |  |  | **<0.001** |  |  |
| Independent |  | — |  |  | — |  |
| Partial assistance |  | 15.2 (6.13 to 37.8) | **<0.001** |  | 8.18 (0.99 to 67.4) | 0.051 |
| Complete assistance |  | 4.12 (0.73 to 23.2) | 0.109 |  | 5.88 (0.13 to 269) | 0.364 |
| Abbreviations: CI = Confidence Interval, OR = Odds Ratio | | | | | | |
| Multivariable model: N=214; Events=42; Non-events=172; AIC=124.7; BIC=171.8; LRT p-value=**<0.001** | | | | | | |


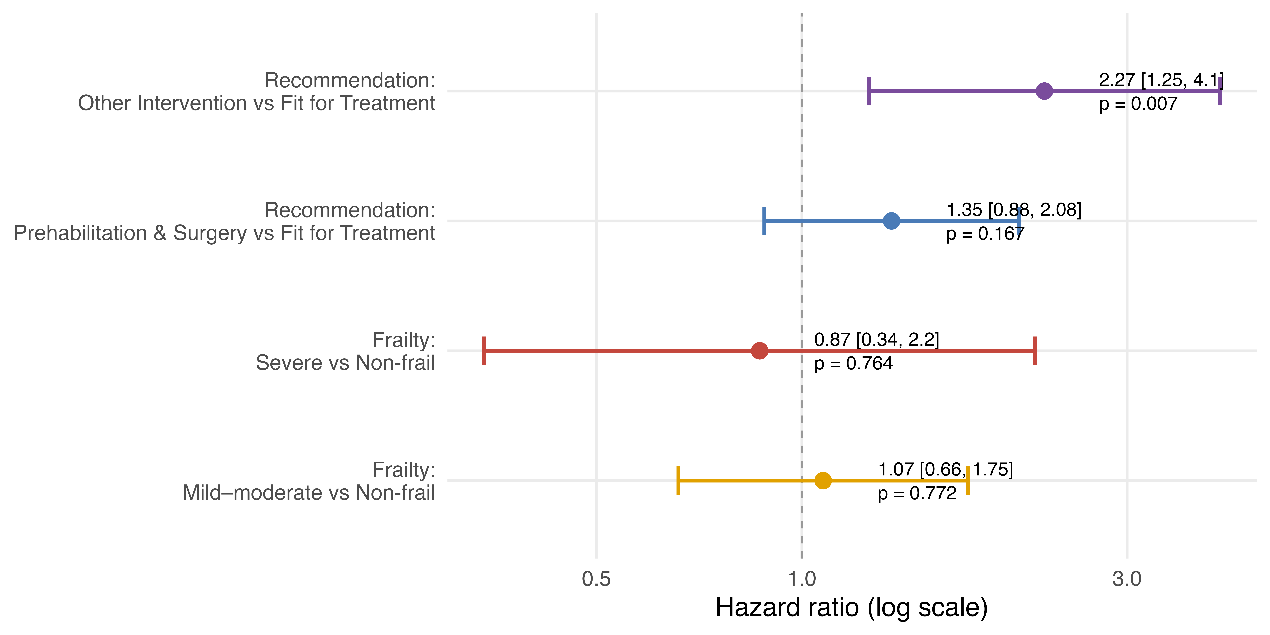


**Supplementary Figure S1. Adjusted hazard ratios for overall survival**.

Multivariable Cox proportional hazards model including frailty category, clinic recommendation, age, gender, Katz ADL, mobility, and G8 category. Compared with patients deemed “Fit for Treatment,” those recommended “Other Intervention” had more twice the hazard of death (HR 2.27 [1.25, 4.1], p = **0.007**). The “Prehabilitation & Surgery” group showed a nonsignificant trend toward higher hazard vs “Fit for Treatment” (HR 1.35 [0.88, 2.08], p = 0.167). After adjustment, frailty category itself (mild–moderate or severe vs non-frail) was not significantly associated with survival. Likelihood ratio tests from nested Cox models showed that adding recommendation to a model with frailty significantly improved model fit (p = **0.029**), whereas adding frailty to a model with recommendation did not (p = 0.844), and there was no strong evidence of frailty–recommendation interaction (p = 0.144).
